# Cerebrovascular injury as a risk factor for amyotrophic lateral sclerosis

**DOI:** 10.1136/jnnp-2015-311157

**Published:** 2015-08-10

**Authors:** Martin R Turner, Raph Goldacre, Kevin Talbot, Michael J Goldacre

**Affiliations:** 1 Nuffield Department of Clinical Neurosciences, Oxford University, John Radcliffe Hospital, Oxford, UK; 2 Nuffield Department of Population Health, Oxford University, Oxford, UK

**Keywords:** ALS, EPIDEMIOLOGY, MOTOR NEURON DISEASE, CEREBROVASCULAR

## Abstract

**Objective:**

To use an unbiased method to test a previously reported association between cerebral arteriovenous malformation (AVM) embolisation and the subsequent development of amyotrophic lateral sclerosis (ALS).

**Methods:**

A hospital record linkage database was used to create cohorts of individuals coded as having cerebral and peripheral vessel AVMs, stroke (separately for haemorrhagic and ischaemic), transient ischaemic attack (TIA) and subarachnoid haemorrhage (SAH). The rate ratio for subsequent ALS was compared to a reference cohort.

**Results:**

An increased rate ratio for ALS was found in relation to prior AVM (2.69; p=0.005), all strokes (1.38; p<0.001), and TIA (1.47; p<0.001).

**Conclusions:**

Cerebrovascular injury from a variety of causes, rather than the presence of AVM or the associated embolisation procedure per se, may be a risk factor for ALS within the context of a more complex multiple-hit model of pathogenesis.

## Introduction

The pathological cascade leading to the fatal adult neurodegenerative disorder amyotrophic lateral sclerosis (ALS) is not yet clear. Cases linked to single genetic mutations are clinically indistinguishable from the vast majority with apparently sporadic disease, and some of the genes linked to hereditary ALS have seemingly disparate functions. It seems, therefore, that ALS involves a final common pathway reached by variable upstream routes.^1^ No strong epidemiological risk factors have been identified for ALS, other than increasing age. Pathogenesis in a relatively rare disease like ALS is, therefore, likely to involve a multiple-hit model.^2^

A retrospective observational study by Valavanis *et al*^3^ identified seven cases of ALS among 1114 patients seen over a 24-year period (with a median follow-up of 11 years), who had undergone embolisation of a cerebral arteriovenous malformation (AVM). Two further anecdotal cases of ALS, occurring 11 and 14 years after the initial embolisation procedure, respectively, were subsequently reported by another centre.^4^ As publication bias is possible in case series, we sought to test this previously unrecognised association in an unbiased fashion using a hospital record-linkage data set and a reference population.

## Methods

An all-England national record-linkage data set of Hospital Episode Statistics and mortality data (1999–2011) was used to undertake studies of cohorts of people with AVM of cerebral vessels (ICD-10 Q28.2), AVM of precerebral vessels (ICD-10 Q28.0) and peripheral AVM (ICD-10 Q27.3) to determine the risks of subsequent ALS relative to a reference cohort. This was repeated for cohorts diagnosed with subarachnoid haemorrhage (SAH) (ICD-10 I60), haemorrhagic stroke (ICD-10 I61-I62), cerebral infarction (ICD-10 I63), and transient ischaemic attack (TIA, ICD-10 G45).

We constructed cohorts of people with each ‘exposure’ condition by identifying every individual in the data set who had a record containing the relevant exposure diagnosis code(s). These individuals were followed up, through record linkage, for any subsequent record of ALS. We restricted ALS outcomes to those diagnosed for 1 year or more after cohort entry to reduce possible bias from misdiagnosis or surveillance. Any individual who had a record of ALS dated earlier than or at the same time as the earliest record of the exposure condition was excluded from the study. For comparison with each exposure cohort, a ‘reference cohort’ was constructed and followed-up in the same way. This cohort comprised over 8.5 million individuals in the data set with no record of ALS—people admitted to hospital for a wide range of mainly minor medical and surgical reasons, including: cataract, inguinal hernia, gallbladder disease, haemorrhoids, varicose veins, internal derangement of knee, deflected septum, nasal polyp, selected disorders of teeth, upper respiratory tract infections, ingrowing toenail and other diseases of nail, sebaceous cyst, otitis externa/media, bunion, dilation and curettage, appendectomy, hip replacement, and knee replacement. We standardised the cohorts by age, sex, year of admission, region of residence, and social class, and censored the person-days of follow-up for death. We calculated expected numbers of people with ALS in each cohort, compared these with the observed number, and calculated standardised rate ratios. Detailed methods are published elsewhere.^5^

The current programme of analysis of the data has been approved by the English National Health Service (NHS) Central Office for Research Ethics Committees (reference number 04/Q2006/176).

## Results

Numbers of people in each cohort are shown in table 1. Of those in the AVM cohort, 76% were aged 15–64 years and 15% were aged 65 years and above. The corresponding figures for the SAH cohort were 64% and 35%, respectively; 82% of the stroke cohort and 76% of the TIA cohort were aged 65 years and over. All comparisons between these cohorts and the reference cohort were age-standardised, (see Methods Section). As table 1 shows, significantly elevated rate ratios for ALS were observed in the cohorts of people with AVM (all types combined, age-standardised rate ratio (RR) 2.69; 95% CI 1.23 to 5.12; p=0.005). Although analysis in relation to AVM subtypes (cerebral, precerebral and peripheral) showed a significant association only for cerebral vessel AVM, the statistical power from the smaller numbers involved was felt to be too low to draw firm conclusions with regard to location. Increased rate ratios for ALS were also found in relation to stroke (either haemorrhagic or ischaemic RR 1.38; 95% CI 1.27 to 1.51; p<0.001), and TIA (RR 1.47; 95% CI 1.29 to 1.68; p<0.001). The significance of the increased rate ratio observed for ALS in the cohorts of people with SAH was equivocal (p=0.053).

**Table 1:** Observed numbers (Obs) patients in the cohorts of people with AVM, SAH, stroke, and transient ischaemic attack, who had a subsequent hospital diagnosis of ALS; expected numbers (Exp) based on comparison with the reference cohort; rate ratios and 95% CI

‘Exposure’ disease	Obs	Exp	RR	95% CI	p Value
All AVM (n=12 220)†	9	3.3	2.69	1.23 to 5.12	0.005*
AVM, cerebral vessels (n=6345)	5	1.7	2.89	1.20 to 6.94	0.035*
AVM, pre-cerebral vessels (n=872)	1	0.3	3.95	0.10 to 22.04	0.623
AVM, peripheral (n=6640)	4	1.7	2.39	0.65 to 6.13	0.158
All stroke (n=1 004 289)†	598	450.8	1.38	1.27 to 1.51	<0.001*
Haemorrhagic (n=179 717)	84	64.8	1.3	1.04 to 1.62	0.019*
Ischaemic (n=526 575)	306	238.2	1.31	1.16 to 1.47	<0.001*
Unspecified (n=389 919)	269	178.2	1.55	1.36 to 1.75	<0.001*
SAH (n=59 227)	28	19.1	1.47	0.98 to 2.13	0.053
Transient ischaemic attack (n=265 676)	243	168.4	1.47	1.29 to 1.68	<0.001*

*p<0.05.

†Total exceeds constituent groups because the same patient may have a different diagnosis at different admissions. In this circumstance, the researchers have no way of knowing which diagnosis is the most appropriate one. The rate ratio is calculated from the formula (O/E) in the exposure cohort divided by (O/E) in the reference cohort. O/E, Observed numbers/expected numbers.

ALS, amyotrophic lateral sclerosis; AVM, arteriovenous malformation; SAH, subarachnoid haemorrhage

## Discussion

Our findings support the published association, albeit small in absolute terms, between the diagnosis of AVM and the subsequent development of ALS, with additional associations noted between ALS and prior non-SAH-related strokes (both ischaemic and haemorrhagic), and TIAs. By considering vascular disease more generally we have previously demonstrated that there is no positive association between ALS and prior ischaemic heart disease.^6^

Only three of the seven AVM cases in the Valavanis *et al* series actually presented with cerebral haemorrhage and no patient was said to have had sustained haemorrhage as a result of the procedure. Instead, the feature identified as common to all cases was “a rare AVM architecture characterised by significant perinidal angiogenesis”, and the authors speculated that the embolisation procedure might be a significant provoking factor. They linked this to a non-significant (p=0.11) reduction in serum of the putatively neuroprotective vascular endothelial growth factor in the patients. Our additional stroke and TIA associations support a much broader common factor for cerebrovascular injury in ALS.

For the 95% of ALS that is apparently sporadic, genetic factors are complex, variable and probably play a much smaller individual role, with additional environmental risk factors contributing to a multiple-hit model.^2^ ALS is typically focal in the location of initial symptoms. A possible association of prior damage to the motor cortex on the subsequent development of ALS was explored in an uncontrolled retrospective observational study, where 18 of 1835 ALS clinic patients had a documented lesion in this area. In four of these, the lesion was an AVM.^7^ In 15 of the 18 cases, the site of first ALS symptoms was contralateral to the lesion-containing hemisphere whereas, in all of the seven AVM cases from Valavanis *et al*, symptom onset was ipsilateral to the lesion. Furthermore, we have reported a case of ALS where the development of symptoms was directly referable to the site of a cerebral aneurysm, with the hypothesis that the associated cerebrovascular injury acted as a nidus for propagation of pathology.^8^ It is possible that other non-vascular focal pathology, for example, cerebral abscess or encephalitis, might act in the same way, though these were not specifically considered in our study due to the limited specificity and range of ICD coding.

We have previously demonstrated that prior hospital admission for a major head injury or physical trauma is not a risk factor for ALS.^9^ This does not, however, exclude other causes of non-traumatic cerebral tissue injury as a contributory factor in the development of ALS in an individual who may be vulnerable for other reasons. Tissue injury might then, in some cases at least, be a final trigger in an ‘ALS-primed’ system. Production of the protein TDP-43, mislocated cytoplasmic aggregates of which are the defining signature common to nearly all cases of ALS, has been noted to be upregulated as part of the normal cellular response to neuronal injury.^10^ Thus, in the light of our findings, we raise the possibility that the activation of cerebral neuronal injury-response pathways, which might occur from a variety of insults, is part of a multiple-hit model of pathogenesis in ALS.
